# Tooth marker of ecological abnormality: The interpretation of stress in extinct mega herbivores (proboscideans) of the Siwaliks of Pakistan

**DOI:** 10.1002/ece3.9432

**Published:** 2022-10-30

**Authors:** Muhammad Ameen, Abdul Majid Khan, Rana Manzoor Ahmad, Muhammad Umar Ijaz, Muhammad Imran

**Affiliations:** ^1^ Institute of Zoology University of the Punjab Lahore Pakistan; ^2^ Department of Zoology, Faculty of Sciences University of Sialkot Sialkot Pakistan; ^3^ Department of Zoology Government College University Lahore Pakistan; ^4^ Department of Zoology, Wildlife and Fisheries University of Agriculture Faisalabad Pakistan

**Keywords:** enamel, hypoplasia, palaeoecology, proboscidea, Siwaliks

## Abstract

Climate affects living ecosystems and defines species physiology. Climate change causes certain stress on animals, recorded as Enamel Hypoplasia (EH). Proboscideans, the mega herbivores, were extensively represented in the Siwaliks of Pakistan between the Middle Miocene to Pleistocene (~15.99–~0.6 Ma). This study was carried out on **15** species from **9** genera and **4** families using **319** teeth from **266** individual quarries. Our results revealed that **20.06% (64/319) of** teeth were infected by EH. Family Deinotheriidae faced higher stress during the terminal of the Middle Miocene **(EH 25%)**. Dental characters of deinotheres indicated that this family preferred soft vegetation like C_3_ plants and failed to survive in grassland ecology at the onset of the Late Miocene (~10–9 Ma). Gomphotheriidae **(EH 21.05%)** and Stegodontidae **(EH 23.40%)** survived through warm and dry climatic conditions of the Late Miocene, but could not survive the cool and dry climate of Plio‐Pleistocene where grasslands were abundant with less browsing activity. Family Elephantidae **(EH 8.47%)** was successful in drier conditions and utilized the exclusive C_4_ diet in open grasslands as efficient grazers, indicated by their tooth morphology. Elephantids were dominant of the proboscideans in open grassland and drier climate during Plio‐Pleistocene in the Indian subcontinent. We assume that change in the Siwalik palaeoenvironment was governed by a microclimate.

## Graphical abstract

Enamel hypoplasia is a tooth marker of ecological abnormality which can also be used in fossil animals to evaluate the likelihood of extinctions. The Siwalik paleoenvironment is being tried to assess by using teeth of the fossil proboscideans.

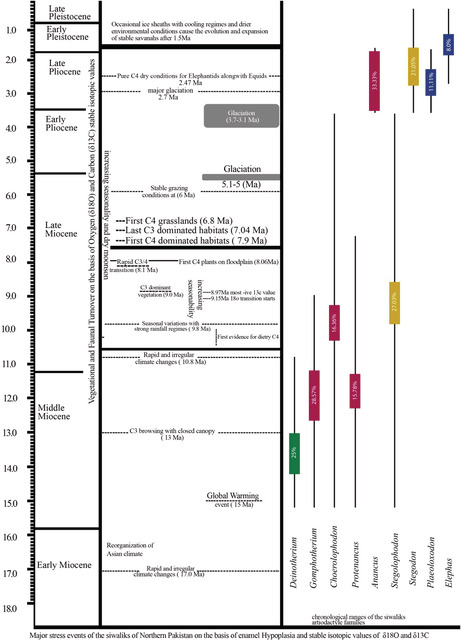

## 1 INTRODUCTION

The Cenozoic geology of Pakistan is the result of a continental collision between the Indian and Eurasian plates. The lower Cenozoic succession of the Greater Indus Basin in Pakistan preserves an important East Tethyan marine succession through the Paleocene–Eocene (Afzal et al., 2011). The Himalayas is well known as the youngest, highest, and one of the best studied continental collision orogenic belts. On a broad scale, the beauty of the Himalayas is to form a relatively simple orogenic belt (Searle & Treloar, 2020). The uplift is started at nearly 60 Ma (Barnes et al., 2011; Beck et al., 1995). This stratigraphic array preserves uninterrupted continental sedimentation along with remains of varied vertebrate fossil groups, especially the mammals. Multiple episodes of rapid and gradual climatic changes influenced the evolution and ecology of mammalian species and communities throughout the Cenozoic (Blois & Hadly, 2009).

The stratigraphy and palaeontology of the Himalayas foreland basin have been studied since the mid‐1800s. The fossil yielding areas of Pakistan are extensive and are among the richest biostratigraphic sequences in the world. It comprises the entire Potwar, sub‐hills of the Himalayas, Kohat, and peaks of D. G. Khan in Punjab, Bugti hills in Baluchistan, Ziarat, Muslim Bagh, southern Bolan, and Dadu in Sindh. Some minute fossil yielding patches are Lasbela and Makran coast. However, the current study purely consists of the Potwar plateau, the Siwaliks of Northern Punjab, Pakistan, having the most frequent occurrences of Proboscidean remnants relatively. Several vertebrate fossil yielding sites called “localities” have been reported in this Potwar plateau. The most promising of these are; Chinji with adjoining areas, Vasnal, Kallar Kahar, Dhok Talian, Phadial, Dial, Padri, Kanhatti, Tatrot, Bhandar, Hasnot, Kotal Kund, Jalalpur, Khalaspur, Rohtas, Dhokawan, Sardhok, Gujar Khan, Dhok Pathan, Dhulian, Kamlial, Darat, Pari Darwaza, Kakrala, Lehri, Ratial, Kundal Nala, Maluwal, Panjan, Dhok Mila, Thathi, Markhal, and Nila.

An overview of the detailed classification narrative of the Siwaliks strata represented by various scientists comprising of major partitions and the correlated sub‐partitions on the basis of lithology is given below in Table 1.

**TABLE 1:** Classification of the Siwaliks (Potwar Plateau) of Northern Pakistan on a lithological basis according to different authors.

Sr. #	Chronology	Partitions of the Siwaliks			Sub‐partitions of the Siwaliks				Timescale
		Colbert (1935) “Series”	Pilgrim (1913) “Divisions”	Danilchik and Shah (1967) “Groups”	Pilgrim (1913)	Anderson (1927), Cotter (1933), Pinfold (1918)	Lewis (1937)	Kravtchenko (1964)	
1	Pleistocene 2.58–0.1 Ma	Upper Siwaliks	Upper Siwaliks	Upper Siwaliks	Boulder Conglomerate zone	Boulder Conglomerate stage	Boulder Conglomerate Formation	Soan Formation	0.6–0.1 Ma Dennell et al. (2006)
					Pinjor zone	Pinjor stage	Pinjor Formation		2.58–0.6 Ma Dennell et al. (2006) Patnaik (2013) Nanda (2015)
	Pliocene 5.33–2.58 Ma				Tatrot zone	Tatrot stage	Tatrot Formation		3.58–3.3 Ma Barry et al. (2002) 5.33–2.5 Ma Barry et al. (2002) 3.6–2.5 Ma Nanda (2013)
2	Late Miocene 11.63–5.33 Ma	Middle Siwaliks	Middle Siwaliks	Middle Siwaliks	Dhok Pathan zone	Dhok Pathan stage	Dhok Pathan Formation	Dhok Pathan Formation	10.1–5.33 Ma Raza (1997) Barry et al. (2002)
					Nagri zone	Nagri stage	Nagri Formation	Nagri Formation	Gradstein et al. (2020) 11.63–9.0 Ma Barry et al. (2002)
3	Middle Miocene 15.99–11.63 Ma	Lower Siwaliks	Lower Siwaliks	Lower Siwaliks	Chinji zone	Chinji stage	Chinji Formation	Chinji Formation	14.2–11.63 Ma Barry et al. (2002)
					Kamlial zone	Kamlial stage	Kamlial Formation	Kamlial Formation	18.0‐14 Ma Barry et al. (2002) Raza (1997) 18.3–14.3 Ma Johnson et al. (1982)

The Elephants are the largest land mammals having herbivorous diet and graviportal limbs. The fossil ancestors of proboscideans were much diverse in body shape, size, and ecological adaptations and dispersed to all continents except Australia (Osborn, 1934). The available data showed that the proboscideans have wide occurrence and great diversity in the localities of the Eocene and Oligocene of Africa and the Arabian Peninsula (Sanders, 2010). Because of morphological diversity, adaptations, variation in diet, and environmental configurations, compared to other mammals, proboscideans remained confined to Africa upto the Late Oligocene times (Sen, 2013). The earliest dispersal of Proboscidea out of Africa was called the “First Proboscidean Datum Event” (Madden & Van Couvering, 1976) and dated back to the Early Miocene from the Bugti Hills of Pakistan (Antoine et al., 2003; Tassy, 1990; Van der Made, 1996; Van der Made & Mazo, 2003) at about 21 Ma ago (Van der Made & Mazo, 2003). In the following events, several proboscidean genera; *Gomphotherium*, *Deinotherium*, *Choerolophodon*, and *Zygolophodon*, occurred in the Eurasian record after the Early Miocene and became well established during the Middle Miocene. In Western Europe, the proboscideans appeared during the Early Burdigalian stage of European land mammal ages, correlated to the Late MN3 zone (Mammal Neogene), which is equal to 16.9 Ma (Antoine et al., 1997). The proboscideans of southeastern Europe were present considerably earlier than in Western Europe. This was around 18.4 Ma (Koufos et al., 2003) earlier in MN3, early Miocene.

The Order Proboscidea Illiger, 1811 has 42 genera and 10 families with 175 species and subspecies (Shoshani & Tassy, 2005). In the Siwalik sub‐region of Pakistan, the order Proboscidea comprises of four Siwalik families, Deinotheriidae Bonaparte, 1841, Gomphotheriidae Hay, 1922, Stegodontidae Osborn, 1918, and Elephantidae Gray, 1821 and 10 valid genera, *Deinotherium* Kaup, 1829, *Gomphotherium* Burmeister, 1837, *Anancus* Aymard, 1855, *Protanancus* Arambourg, 1945, *Choerolophodon* Schlesinger, 1917, *Paratetralophodon* Osborn, 1929, *Stegolophodon* Schlesinger, 1917, *Stegodon* Falconer, 1857, *Palaeoloxodon* Matsumoto, 1924, and *Elephas* Linnaeus, 1758 with 22 extinct species from which 18 species are valid (Tassy, 1983, 1996) and 4 are disputed with unsettled taxonomy. The present study included all the 4 Siwalik families along with 9 genera and 15 species. The species with less than 4 tooth samples have been excluded from this study for the analysis of Enamel Hypoplasia (EH).

### 1.1 Palaeogeography of proboscidean

**FIGURE 1:**
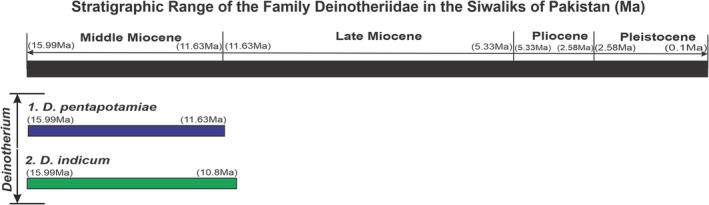
Indicating the stratigraphic range of the family Deinotheriidae in the Siwaliks of Pakistan (Khan et al., 2011; Patnaik, 2013).

The Deinotheriids were characterized by the presence of vertically erupted all check teeth and downwardly pointed lower incisors (tusks). This is a monophyletic Siwalik group with only a single genus, *Deinotherium* (Sarwar, 1977). The genera *Prodeinotherium* Éhik, 1930, and *Antoletherium* Falconer, 1868 cannot be differentiated from *Deinotherium*. The taxonomic status of the genus is still controversial, and some authors (Tiwari et al., 2006) attributed the smaller sized animals as *Prodeinotherium*. However, the current study of EH is based only on two valid species of the genus *Deinotherium*; (i) *D. pentapotamiae* and (ii) *D. indicum* from the Siwaliks of Pakistan, given in Figure 1. These two species have overlapping temporal range, but they were different in body size, presence of tubercles, and very prominently developed talon ridge in M_2_ (second molar) of *D. indicum*, whereas these characteristics were absent in *D. pentapotamiae* (Sahni & Tripathi, 1957). These dental assemblages belong to the Middle Miocene Siwaliks of Northern Pakistan (Sarwar, 1977).

**FIGURE 2:**
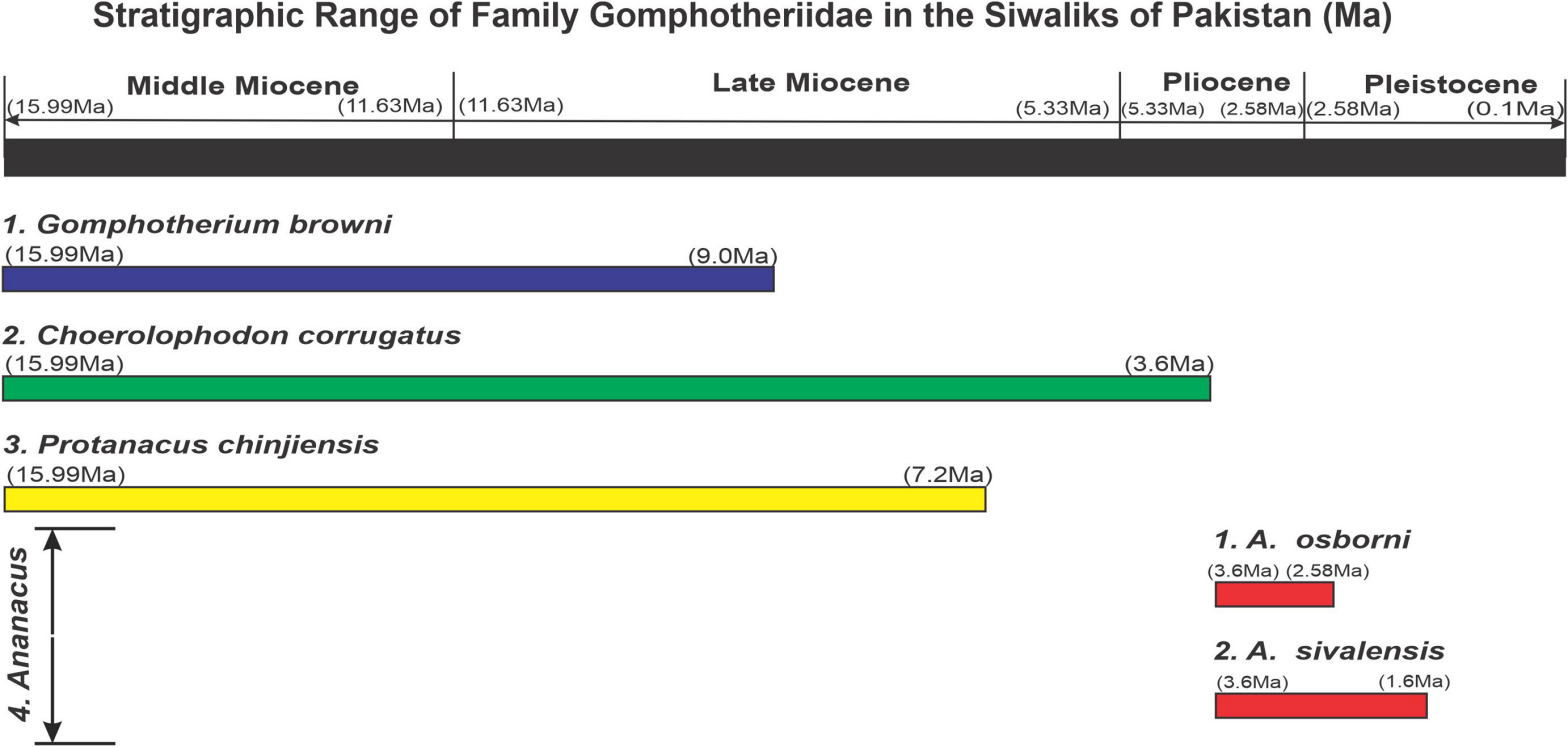
The stratigraphic range of genera of the family Gomphotheriidae in the Siwaliks of Pakistan (Abbas et al., 2018; Behrensmeyer & Barry, 2005).

The gomphotheres were the proboscideans known by their fossil record from all over the globe except from Antarctica and Australia from the Early Miocene to Late Pleistocene. By general appearance, they had two pairs of oppositely pointed upper and lower tusks (Lambert, 1992). They are characterized by the presence of trefoil shaped wear pattern (Prado & Alberdi, 2008). The earliest gomphotheres appeared in the Early Miocene (approx. 22 Ma) of Africa and later on dispersed into Asia and Europe (Tassy, 1996). These proboscideans are characterized by 3 – plated first two molars having three cusp pairs, and because of this character, these are known as trilophodonts. The evolution of the gomphotheres is considered to be the second radiation after the ancestral radiation of proboscideans. Molars of *A. osborni* are more progressive than *A. sivalensis* in shape (strongly oblique ridge‐plates) and a number of ridge‐plates (Sarwar, 1977). The palaeogeography of the Siwalik gomphotheres is given in Figure 2.

There is a debate on the taxonomical status of the stegodons, either they form a separate family or belong to Elephantidae (Saegusa et al., 2005; Shoshani & Tassy, 1996). This Siwalik clade consists of two genera, *Stegodon* and *Stegolophodon*, which are considered to have evolved from the Gomphotheriidae. The stegodons have low crowned teeth with many plate‐like ridges in contrast to the high crowned plated molars of elephants and mammoths. In general, *Stegolophodon* has four tusks, two on each jaw, while *Stegodon* had only two straight tusks in the upper jaw. The earliest *Stegolophodon* fossils belong to the Middle Miocene of Thailand from there, the genus spread throughout the northern hemisphere, which may have happened in response to the Mid‐Miocene climatic warming (Saegusa, 1996). *Stegodon* is assumed to have originated from *Stegolophodon* in South China during the Early Pliocene because of the higher number and diversity in this region. *Stegodon* remains were also reported from Kenya, and Africa dated back to 6.5 million years (Saegusa, 1996). *Stegodon* has also been known from Japan with several endemic species (Saegusa et al., 2005). In the Siwaliks of Pakistan, the chronological record of *Stegolophodon* is from the Middle Miocene to Pliocene (15.99–3.6 Ma), whereas *Stegodon* was found from the Pliocene to Pleistocene (3.6–0.6 Ma) epochs. This chronological record of stegodons may support the hypothesis of their origin from *Stegolophodon*. Apomorphous characters, and forward inclination of the occiput and basilar tubercles are missing in *S. bombifrons* but are shared by *S. insignis* and *S. ganesa*. Only the lectotype fits to *S. bombifrons*. Some former palaeontologists (Hooijer, 1955; Lydekker, 1876; Osborn, 1942) have proposed that *S. ganesa* is the junior synonym of *S. insignis*, regardless of the fact that both species have considerable differences in the cranial morphology.

**FIGURE 3:**
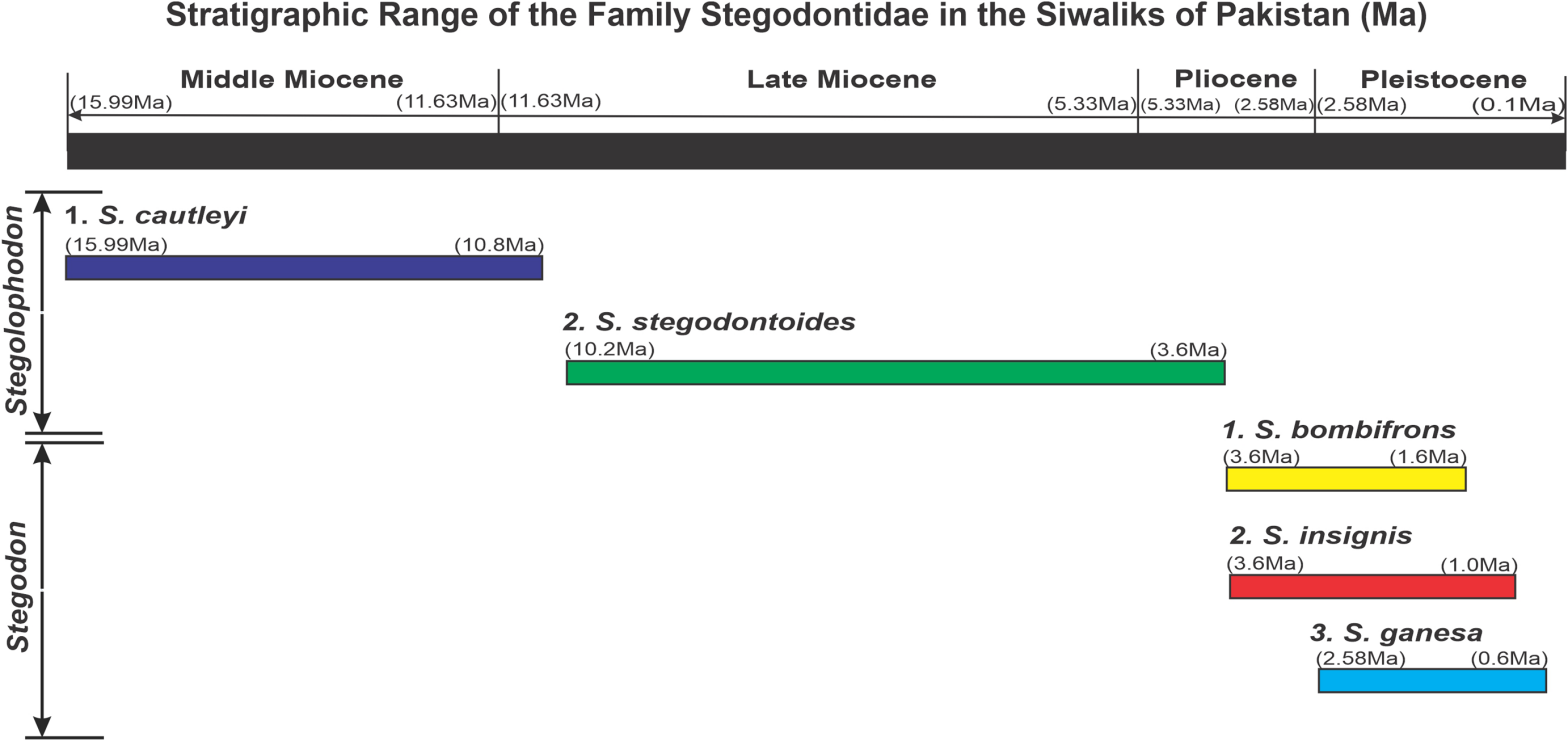
The stratigraphic range of genera of the family Stegodontidae in the Siwaliks of Pakistan (Osborn, 1942; Saegusa, 1987; Saegusa et al., 2005; Tassy, 1983).

On the basis of both sexual dimorphism and ontogenetic changes in cranial features, *S. insignis* and *S. ganesa* are particularly dissimilar (Abbas et al., 2019), contrary to some previous authors, claimed as a single collective species (Chauhan, 2008). We concluded that *S. insignis* and *S. ganesa* are not a single collective species (Saegusa, 1987). The palaeogeography of the Siwalik Stegodontids is given in Figure 3.

**FIGURE 4:**
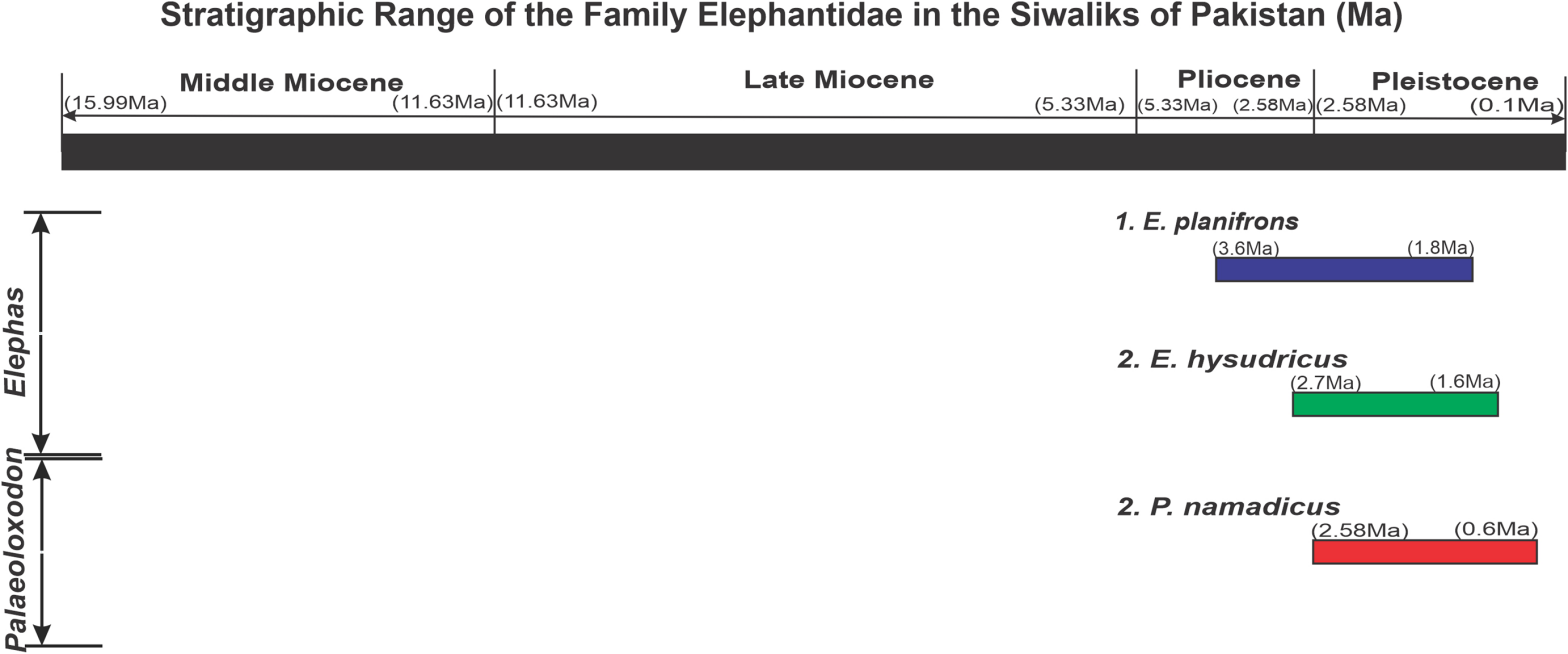
The stratigraphic range of different members of the family Elephantidae in the Siwaliks of Pakistan (Agarwal et al., 1993; Dennell et al., 2006; Hussain et al., 1992; Nanda, 1997).

Elephantids are the largest proboscideans that roam on earth. Usually, a very large pair of straight tusks were present only in the upper jaw of the animal. The tooth replacement patterns and plated structure of dentition are much similar to the family Stegodontidae, indicate their relationship. These immigrants had originated and diversified during the Pleistocene of the Siwaliks. The fossil remains of *Palaeoloxodon* were found from the Pleistocene deposits of Asia, East Asia, and Europe. *Palaeoloxodon namadicus*, the largest terrestrial mammal ever on Earth (Larramendi, 2015), is thought to have become extinct during the Late Pleistocene of the Siwaliks. Shoshani and Tassy (2005) recognized seven genera (*Stegotetrabelodon*, *Stegodibelodon*, *Primelephas*, *Loxdonta*, *Palaeoloxodon*, *Elaphas*, and *Mammuthus*) of the family Elephantidae. *Loxodonta* and *Elephas* are the living genera from Africa and Asia, respectively. In the Siwaliks of northern Pakistan, *Palaeoloxodon* and *Elephas* are known by three extinct species, *P. namadicus*, *E. planifrons*, and *E. hysudricus*. The stratigraphic range of the family in the Siwaliks of Pakistan is from Pliocene to Pleistocene (3.58–0.6 Ma) is given in Figure 4.

### 1.2 Enamel Hypoplasia

**FIGURE 5:**
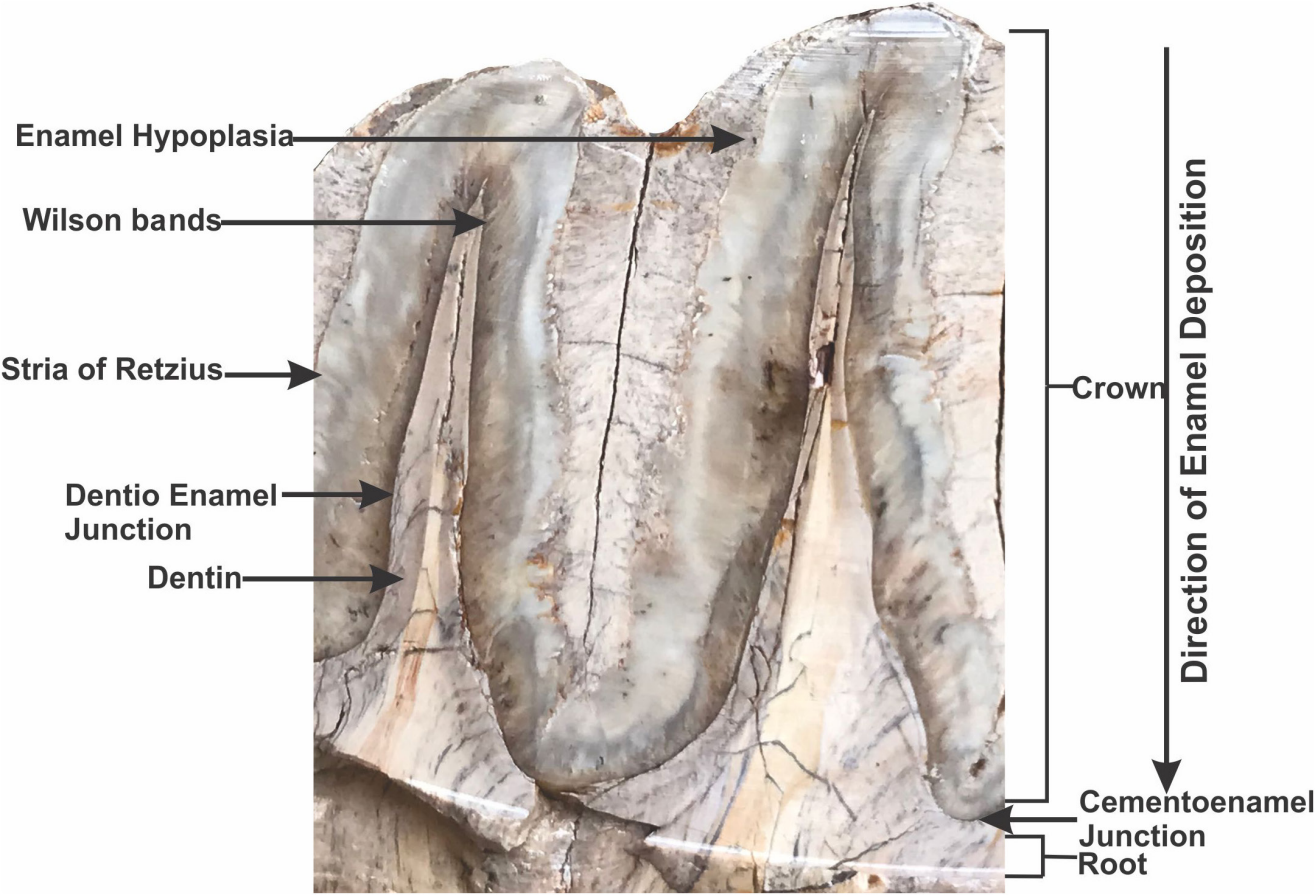
Longitudinal cross section of mammalian (proboscidean) tooth for the development of enamel and dentine, a diagrammatical representation of the formation of EH.

Enamel, the whitish casing of the tooth crowns, is highly mineralized and the most durable tissue in the mammalian body (Kierdorf et al., 2012; Shawashy & Yaeger, 1986). Enamel Hypoplasia (EH) is a deficit in tooth enamel due to the physiological offenses that cripple ameloblasts deposition during the secretory phase of amelogenesis (Guita, 1984; Sarnat & Schour, 1941; Shafer et al., 1983; Yaeger, 1966). The ameloblasts, tissues which make enamel, may describe its great degree of sensitivity to physiological perturbations. The EH is ensured when the stress intensity reaches a certain threshold level, and if the ameloblasts are active at that time of physiological stress, then the ameloblasts can function no longer. This stress threshold may be passed in the chronic or episodic manner (Goodman & Rose, 1990). However, EH is a permanent failure of tooth enamel (Figure 5) to attain its normal thickness during the development of the tooth crown (Goodman et al., 1987; Goodman & Rose, 1991). The anomaly may appear as a horizontal or vertical groove more or less encircling the tooth crown or as discrete “pin prick” sized cavities in the ordinary enamel figure, presumed to correspond to less severe stress (Ainamo et al., 1982). It can be associated with a myriad of causes but is generally linked to malnutrition, infections, or febrile disease (Suckling, 1989).

Tooth enamel, in this regard, is unique because of its inability to remodel. On the basis of ease of enamel examination, sensitivity, failure to remodel, and a chronological array of its developmental pattern, it can be the perfect tissue for recording alterations in an animal physiology during its tooth development (Kreshover, 1940; Massler et al., 1941; Sarnat & Schour, 1941). The EH in fossilized teeth has a potential of providing a perspective into the ecological conditions of an extinct animal's life. The presence of EH in fossilized teeth has been recently unveiled by many paleontologists (Ahmad et al., 2018; Barrón‐Ortiz et al., 2019; Bohmer & Rossner, 2017; Byerly, 2007; Mead, 1999; Odendaal et al., 2003, 2004; Roohi et al., 2015), added additional and much reliable evidence to propose the palaeoclimatic fluctuations on a regional scale.

The beauty of the present framework is the application of EH and selection of such specific taxon of vertebrate mammals (Proboscidea) whose fossil assemblages cover the whole chronological range of the Siwaliks from Middle Miocene to Late Pleistocene (15.99–0.6 Ma). The analysis of EH in fossil proboscideans and their correlation with the Neogene environmental conditions was performed with the following objectives; (i) to know which taxon of the Siwalik proboscideans was influenced more severely by the changing palaeoecological conditions, (ii) to document the type of systemic stress they faced and their ecological response to the vegetational pattern and success rate of different taxa, (iii) to compare the occurrence of the frequency of EH in proboscideans with other Siwalik mammals to understand their response to the changing paleoecological conditions.

### 1.3 Abbreviations

EH, Enamel Hypoplasia; PUPC, Punjab University Palaeontological Collection; UZ, University of the Punjab, Department of Zoology (old Abbreviation); P1, First premolar; P2, Second Premolar; P3, Third Premolar; P4, Fourth Premolar; dP, Deciduous Premolar M_1,_ First Molar; M_2_, Second Molar; M_3_, Third Molar. The uppercase alphabet represent the upper (maxillary) dentition, and the lowercase represent the lower (mandibular) dentition as; M2 and m2.

## 2 MATERIALS AND METHODS

### 2.1 Collection and storage of proboscidean samples

Three hundred nineteen proboscidean teeth, either isolated or in mandibles, from 266 quarries were analyzed for EH. These include newly and previously collected specimens. Most of the previously used material was surveyed and analyzed. Both fresh and previously collected specimens are being stored in Dr. Abu Bakar Fossil Display and Research Centre, University of the Punjab, Lahore, and Palaeontological Museum and Collection Centre of University of the Punjab, Jhelum Campus, Jhelum, Pakistan. These specimens were macroscopically examined for the presence or absence of EH. The studied fossil material belongs to **4** families, **9** genera, and **15** species of the Siwalik proboscideans.

### 2.2 Identification of specimens

The dental terminology of Tassy (1996) and Harris (1976) was used to identify proboscidean tooth remains. Some old samples with unsettled or disputed taxonomy were also adjusted in the suitable groups with the help of Sarwar (1977), Shoshani and Tassy (1996, 2005), and the unpublished PhD thesis of Dr. Syed Ghyour Abbas et al. (2018). Before the analysis of EH, fossil remains were first scrutinized as “readable” or “unreadable” teeth, and only the readable teeth were used. Teeth were considered unreadable because: (i) extremely fragmented or worn enamel; (ii) being completely covered with dental calculus or cementum; (iii) or their enamel surfaces were impossible to examine, because they were erupting or concealed in the alveolus. Teeth with any of the completely readable sides were recorded. All types of check teeth were included.

### 2.3 Preparation of specimens

The readable dental material was assembled, if broken at some parts, washed, and cleaned before the examination. The specimens whose catalog numbers were vague or wiped out were also included by giving them a new catalog number serially starting from its family alphabet name. For example, a specimen with an erased catalog number, i.e., *Anancus sivalensis*, a member of the family Gomphotheriidae, was written as “PUPC‐G_1_” and so on serially.

The EH analyses were accomplished macroscopically following the methods outlined by Goodman and Rose (1990) and Lukacs (1989, 1999). Different laboratory equipment and scientific instruments were used for the examination and recording of EH as;

- Digital Vernier Caliper with 100 mm measuring capacity.
- Digital handheld HDR camera for photography.
- Large dissecting trays with cotton bed to shift the samples.
- A 60–100 Watt incandescent light, with variable power, was used for laboratory examination of samples.
- A 10× hand held lens was used for magnification, identification, precision, and confirmation of EH.

Each specimen was judged with a 10× hand lens after the examination with the naked eye to rectify the presence or absence of EH. Berti and Mahaney (1995) and Hillson and Bond (1997) also defined that the analyses of EH requa ire detailed examination of each tooth surface under magnifications.

### 2.4 Scoring of Enamel Hypoplasia

During the examination, the position of EH was recorded, starting from root crown junction (RCJ) to enamel defect. The distance was measured to the center of the enamel defect. For each enamel defect, the distance indicated the timing of EH formation. Only labial and lingual surfaces of tooth crowns were contemplated for the occurrences of EH, and only the mandibular and maxillary teeth were used. The lingual and labial surfaces were designated as pretrite (lingual side in upper molars/labial side in lower molars) and posttrite (labial side in upper molars/lingual side in lower molars) on the basis of wear pattern in proboscideans to identify the position of any tooth in jaw. The measurement of EH defects taken in millimeters (mm) relied on the method of Mead (1999) using a digital Vernier Caliper. A mean of three repeated readings was used. The Canon EOS‐350D digital professional series was used to snapshot the EH impression. A maximum of three incidences of EH were recorded on a single tooth as “**m**_**3**_” of *Anancus sivalensis* (Catalogue # PUPC‐G_12_), *Elephas planifrons* (Catalogue # PUPC‐E_1_), and *Palaeoloxodon namadicus* (Catalogue # PUPC‐14/60) Table 2.

**TABLE 2:** Incidences of Enamel Hypoplasia in the Siwalik proboscideans from the Middle Miocene to Pleistocene having an age of 15.99–0.6 Ma approximately.

Siwalik families	Species	Specimen	Locality	Age (Ma)	Enamel Hypoplasia		
					Tooth	Loph/lophid	Location
Deinotheriidae	*Deinotherium indicum*	PUPC‐09/116b	Lava Chinji (Jhelum)	12	m2	2nd lophid	One EH 10.4 mm above the Root Crown Junction (RCJ)
					m3	1st lophid	One EH 7.21 mm above RCJ
		UZ‐66/815	Dial (Jhelum)	13.5	M2	1st loph	One EH 10.20 mm above RCJ
		UZ‐D_1_	Chinji village (Jhelum)	13	m1	3rd lophid	Two EH 10.33 mm above RCJ 26.50 mm above RCJ
		UZ‐66/125	Bhelomar Upper Chinji	13.8	P3	Labial	One EH 8.86 mm above RCJ
	*Deinotherium pentapotamiae*	UZ‐66/41	Bhilomar Chinji Horizon	13.8	P4	Labial	One EH 10.0 mm above RCJ
		UZ‐96	Chinji village (Jhelum)	13	P3	Labial	One EH 10.34 mm above RCJ
		UZ‐67/467	Chinji village	13	M3	1st Loph	One EH 13.86 mm above RCJ
						2nd Loph	One EH 12.22 mm above RCJ
		UZ‐84/95	DBAK (Chakwal)	11.8	m1	1st and 2nd Lophid	One EH 3.15 mm above RCJ
		PUPC‐15/01	Chabbar Syedan	14.2	p4	2st lophid	One EH 5.11 mm above RCJ
					m1	1st lophid	One EH 15.1 mm above RCJ
		PUPC‐15/253	Chabbar Syedan	14.2	m1	2nd & 3rd lophid	Two EH's 6.50 mm above RCJ 9.80 mm above RCJ
					m2	2nd lophid	One EH 19.60 mm above RCJ
Gomphotheriidae	*Gomphotherium browni*	PUPC‐15/14	Kanhatti	12.5	m1	2nd lophid	One EH 12.07 mm above the RCJ

				m1	1st lophid	One EH 5.52 mm above the RCJ

	PUPC‐15/218	Chabbar Syedan	14.2	M3	2st loph	One EH 17.94 mm above the RCJ

	UZ‐67/80	Pari Darweza (Kamlial Formation)	15	M1	1st loph	One EH 07.80 mm above the RCJ

	PUPC‐15/06	Chabbar Syedan	14.2	m3	2nd and 3rd lophid	Two EH's 10.53 mm and 11.78 mm above the RCJ

	UZ‐68/810	Nagri	9.2	M2	1st loph	Two EH's 5.04 mm and 12.97 mm above the RCJ

	UZ‐66/49	Dhok Pathan	6.8	m3	1st lophid	One EH 40.24 mm above the RCJ

	UZ‐67/227	Chinji	13	m3	Last lophid	One EH 22.10 mm above the RCJ

*Choerolophodon corrugatus*	PUPC‐14/39	Dhok Pathan	6.8	M1	2nd loph	One EH 5.02 mm above RCJ

				M2	3rd loph	One EH 10.12 mm above RCJ

	UZ‐67/118	Dhok Pathan	6.8	dp4	1st lophid	Two EH's 5.04 mm and 12.97 mm above RCJ

	PUPC‐15/05	Markhal (Dhok Pathan)	7.3	m3	3rd lophid	Two EH's 16.20 mm and 21.07 mm above RCJ

	PUPC‐15/239	Dhok Mila	9.2	M2	1st and 3rd loph	Two EH's 36.16 mm, 10.96 mm, 5.0 mm, above RCJ

	UZ‐69/627	Vasnal, Bhelomar	12	m3	1st lophid	One EH 17.03 mm above RCJ

	UZ‐68/816	Kanhatti	12.5	M2	2nd last loph	One EH 23.94 mm above RCJ

	UZ‐84/116	Lava, Dhok, Rehmat Ali	11.8	M2	Last loph	One EH 37.16 mm above RCJ

	PUPC‐15/204	Kanhatti	12.5	M1	2nd loph	Two EH's 2.0 mm and 3.57 mm above RCJ

*Protanancus chinjiensis*	UZ‐69/626	Pari Darweza	14	m3	3rd lophid	One EH 21.22 mm above RCJ

	UZ‐47	Chinji bed (Chakwal)	13.8	M3	2st loph	One EH 34.50 mm above RCJ

	PUPC‐15/16	Chabbar Syedan	14.2	m2	4th lophid	One EH 3.57 mm above RCJ

*Anancus osborni*	PUPC‐G_1_	Tatrot zone	3.5	M3	5th loph	One EH 33.0 mm above RCJ

*Anancus sivalensis*	UZ (71/56)	Dhok Awan Tatrot zone Jhelum	3	m3	Last lophid	Two EH's 7.90 mm 17.88 mm above RCJ

	PUPC‐(10/75)	Rohtas	1.6	m3	3rd lophid & Last lophid	Two EH's 27.64 mm above RCJ 31.0 mm above RCJ

	PUPC‐G_12_	Rohtas	1.6	m3	2nd last lophid and Last lophid	Three EH's 42.0 mm, 32.60 mm and 34.30 mm above RCJ
Stegodontidae	*Stegolophodon stegodontoides*	UZ‐86/295	Dhok Pathan	6.8	P4	Lingual	One EH 18.04 mm above RCJ

				M1	labial	One EH 8.42 mm above RCJ

	UZ‐63	Dhok Pathan	6.6	M3	6th Loph	One EH 22.12 mm above RCJ

	UZ‐85/204	Dhok Pathan	6.8	M2	2nd loph 3rd loph	Two EH's 12.95 mm and 25.0 mm above the RCJ

	UZ‐70/22	Dhok Pathan	7	m3 m3	2nd lophid 2nd last lophid	Two EH's 18.70 mm, 24.92 mm on right m3 and One EH 32.0 mm on left m3 above the RCJ

	UZ‐69/06	Lehri	9	M3	3rd, and 5th loph	Two EH's 7.60 mm and 29.5 mm above RCJ, respectively

	UZ‐68/793	Bhandar	6.6	m3	Lingual	One EH 4.58 mm above RCJ

*Stegolophodon cautleyi*	PUPC‐15/250	Kanhatti	12.5	M2	6th loph	One EH 12.13 mm above RCJ

				M2	7th loph	One EH 23.16 mm above RCJ

*Stegodon bombifrons*	UZ‐69/10	Kakrala	3.2	M2	Last loph	One EH's 18.15 mm above RCJ

	UZ‐68/812	Kakrala	3.2	M3	3rd loph	One EH 11.88 above RCJ

				M3	3rd & ^4th^ loph	Two EH's 14.18 and 32.0 mm above RCJ

	PUPC‐14/56	Sardhok	2.4	M3	5th loph	One EH 8.56 mm above RCJ

	UZ‐67/470	Sardhok	2.4	m1	3rd lophid	One EH 15.22 mm above RCJ

	UZ‐10/85	Sardhok	2.4	M3	2nd loph	One EH 3.52 mm above RCJ

*Stegodon insignis*	UZ‐71/61	Kakrala	3.2	m2	4th lophid	Two EH's 34.24 mm and 40.0 mm above RCJ

	PUPC‐S_4_	Kakrala	3.2	m2	4th lophid	One EH 12.50 mm above RCJ

	UZ‐66/03	Panjan	2.3	M2	5th loph	One EH 13.16 mm above RCJ

*Stegodon ganesa*	UZ‐68/792	Sardhok	2.4	M3	4th loph	One EH 38.0 mm above the RCJ

	PUPC‐07/92	Sardhok	2.4	M3	Last loph	One EH 38.0 mm above the RCJ

	PUPC‐S_8_	Panjan	2.3	m2	4th lophid	One EH 17.38 mm above the RCJ
Elephantidae	*Elephas planifrons*	PUPC‐E_1_	Sardhok (Tatrot zone)	3.2	m3	2nd and 3rd last lophids	Three EH's 11.15 mm, 20.26 mm & 32.0 mm above RCJ
		UZ‐70/15	Tatrot	3.5	M3	Middle loph	One EH 47.50 mm above RCJ
	*Elephas hysudricus*	UZ‐68/769	Sardhok (Tatrot zone)	2	M1	Last loph	One EH 26.0 mm above RCJ
		PUPC‐E_2_	Kakrala	3.2	M3	4th and 5th lophs	Two EH's 14.0 mm and 16.55 mm above RCJ
	*Palaeoloxodon namadicus*	PUPC‐14/60	Sardhok	1.8	m3	4th, 5th, and 9th loph	Three EH's 28.28 mm and 35.80 mm and 47.0 mm above RCJ

*Note*: Lowercase letter = Mandibular tooth & uppercase = Maxillary tooth in “tooth” column.

### 2.5 Collective evaluates of EH by two raters

Firstly, two different light sources (sunlight and 60–100 watt incandescent light with variable settings) were used, and the specimens were oriented obliquely, when entailed to the artificial light source following Lukacs (1989, 1999). Several times a variable amount of light appeared greatly useful for individual samples to identify either EH was present or not. The orientation of specimens really matters because EH is sometimes masked by the mirrored light or by the color of the specimen, which shades the tooth anomaly. The analysis was accomplished duly by two observers to create more objective results following Guatelli‐Steinberg (2003). The repeatability and reliability is an indispensable exercise during the examination of EH, especially in proboscidean teeth, because of enamel rugosity and the presence of perikymata. The guidelines by Landis and Koch (1977) were followed for the interpretation of Kappa.

### 2.6 Statistical analyses

Cohen (1960) “Kappa” statistics was run for the explanation of agreement or disagreement between two observers. The calculations of the “*K*” value indicate the inter‐rater reliability between the observers. The values of “*p*” indicate the precision and validity of results, whether the difference in opinion between the two raters is significant or not. Chi‐square test was also performed for paired comparisons. In pursuit of multiple comparisons of EH between each of the families and genera, the Mann–Whitney *U* test, was run after the Kruskal–Wallis test where the comparisons of significant level (*p*‐value) for the occurrence of EH within 4 families and 9 genera were individually calculated. Table 2 provides a complete description of the presence of EH in the Siwalik proboscidean specimens.

## 3 RESULTS

**FIGURE 6:**
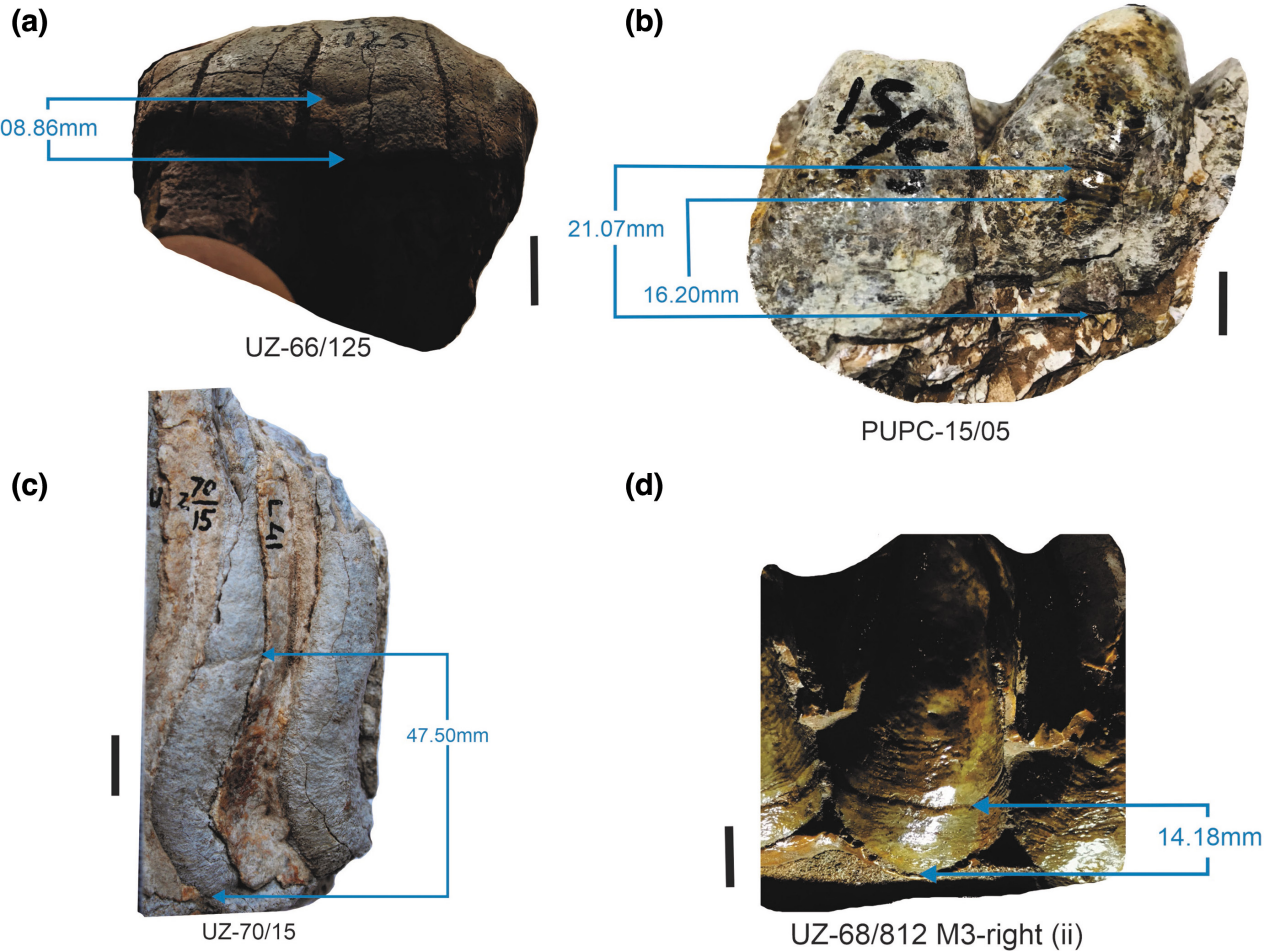
The pictographic representation of selected molars from the Siwalik Proboscidean families showing enamel hypoplasia where the specimen a = Deinotheriidae, b = Gomphotheriidae, c = Elephantidae, and d = Stegodontidae. The marks and measurements of EH are indicated by an arrow in millimeters from the root crown junction. Scale bar of 10 mm is used.

Out of 319 teeth, sixty–four of the 319 teeth analyzed (20.06%) were affected by EH. The highest EH occurrence (25%) was found in the family Deinotheriidae. And the lowest EH occurrence (8.47%) was found in Elephantidae. Generic comparisons would be more accurate where the exact possible stratigraphic range of the taxon can be determined. Generic comparisons indicated that *Anancus*, a Plio‐Pleistocene genus, was found with the highest occurrence (33.33%) of EH, whereas, *Elephas*, having a similar stratigraphic range, was found to be least affected with only 8% occurrence of EH. Table 3 delivers detailed information on the occurrence of EH at the taxon level. Figure 6 imparts the pictographic representation of some representative tooth specimens of the Siwalik proboscideans showing EH.

**TABLE 3:** Proboscidean fossil tooth specimens with percentage prevalence of EH by species, genus, and family.

Family	Genus	Species	Teeth	Teeth with EH	%age of EH by species	%age of EH by genus	%age of EH by family
Deinotheriidae	*Deinotherium*	*D. pentapotamiae*	25	8	32%	25%	25%
		*D. indicum*	27	5	18.52%		
Gomphotheriidae	*Gomphotherium*	*G. browni*	28	8	28.57%	28.57%	21.05%
	*Choerolophodon*	*C. corrugatus*	55	9	16.36%	16.36%	
	*Protanancus*	*P. chinjiensis*	19	3	15.79%	15.79%	
	*Anancus*	*A. osborni*	4	1	25.00%	33.33%	
		*A. sivalensis*	8	3	37.50%		
Stegodontidae	*Stegolophodon*	*S. stegodontoides*	27	8	29.63%	27.03%	23.40%
		*S. cautleyi*	10	2	20%		
	*Stegodon*	*S. bombifrons*	29	6	20.69%	21.05%	
		*S. insignis*	19	3	15.79%		
		*S. ganesa*	9	3	33.33%		
Elephantidae	*Elephas*	*E. planifrons*	26	2	7.69%	8%	8.47%
		*E. hysudricus*	24	2	8.33%		
	*Palaeoloxodon*	*P. namadicus*	9	1	11.11%	11.11%	
	Total		319	64	20.06%		

### 3.1 EH prevalence in Deinotheriids

The results for the frequency of EH by tooth are summarized in Table 3. An overall of 13 out of the total 52 studied teeth of the family Deinotheriidae showed 25% of EH. The species *D. pentapotamiae* comprises of 25 analyzed teeth, out of which 8 teeth hold 32.0% of EH anomaly, whereas, *D. indicum* contributed 5 defected teeth out of 27 and revealed 18.52% of EH (Table 3).

### 3.2 EH prevalence in Gomphotheres

The results for the frequency of EH by tooth are summarized in Table 3. An overall 24 teeth out of the total 114 were found to be affected, which showed a 21.05% occurrence of EH. The individual teeth from five species, *Gomphotherium browni*, *Choerolophodon corrugatus*, *Protanancus chinjiensis*, *Anancus osborni*, and *Anancus sivalensis*, belonging to the four genera of the Siwalik family Gomphotheriidae were showed 28.57%, 16.36%, 15.79%, 25.00%, and 37.50%, frequency of EH, respectively (Table 3). The analysis showed that the lowest number of affected teeth was found from *Protanancus chinjiensis* (15.79%) and *Choerolophodon corrugatus* (16.36%), whereas *Anancus sivalensis* (37.5%) was spotted with the highest number of defected teeth. The results for the incidences of EH in teeth of gomphotheres indicate that *Anancus sivalensis* was affected more severely by the environmental stress compared to the other members of the family. Maximum occurrences of three EH were recorded on a single tooth which indicate that the animal faced multiple episodes of stress during its life history.

### 3.3 EH prevalence in Stegodontids

The present analysis of the family Stegodontidae consisted of 94 teeth examined for the occurrence of EH. Twenty two teeth out of 94 revealed almost 23.40% of EH.

Table 3 provides complete information about the prevalence of EH by the tooth in the two genera, *Stegolophodon* and *Stegodon*, of the family Stegodontidae with five extinct Siwalik species: *Stegolophodon stegodontoides*, *Stegolophodon cautleyi*, *Stegodon bombifrons*, *Stegodon insignis*, and *Stegodon ganesa*. The occurrences of EH were inconsistent in teeth of all the five Siwalik stegodonts (Table 3). The *S. stegodontoides*, a Late Miocene species, and the *S. ganesa*, a Pleistocene species, revealed the highest defected teeth with 29.63% and 33.33% of the occurrence of EH, respectively. The *S. insignis*, a Pliocene species, showed the lowest (15.79%) occurrence of EH. The generic analysis showed that the genus *Stegolophodon* revealed a little bit higher occurrence (27.03%) of EH compared to *Stegodon* (21.05%) without any significant differences.

### 3.4 EH prevalence in Elephantids

The current analysis consisted of 5 defected teeth out of 59 of the family Elephantidae with 8.47% occurrence of EH. The family comprises of two genera, *Elephas* and *Palaeoloxodon*, with three species, *E. planifrons*, *E. hysudricus*, and *P. namadicus*, having 7.69%, 8.33%, and 11.11% frequencies of EH, respectively. The Plio‐Pleistocene occurring genus *Elephas* contained 4 affected teeth out of 50, indicating 8.0% of EH, whereas, the Pleistocene genus, *Palaeoloxodon*, showed 11.11% of EH anomaly with only 1 defected teeth out of 9 (Table 3).

### 3.5 An inter‐rater reliability: interpretation of Kappa

The data from two individual raters were compared to check the reliability in two diagnosing opinions in the samples of the family Deinotheriidae. There was almost substantial agreement between the two diagnoses, *K* = 0.749 and *K* = 0.71 for samples of the family Deinotheriidae by using Cohen's Kappa statistics analyzed in natural and artificial lights, respectively, and there was a nonsignificant difference (*p* > .05) in opinion between the two raters. The *p*‐value at some points indicates the strength of disagreement between the raters, which specifies the difficulty in the evaluation of EH in the Deinotheriids. The trouble in diagnosis was encountered because the tooth enamel of the Deinotheriids is supplementary rugose which is somehow capacitated by another rater. The samples were examined in both kinds of lights to get maximum accuracy. A perfect agreement was found by the Kappa results, which showed that *K* = 0.898 and *K* = 0.874 for samples of family Gomphotheriidae analyzed in natural light as well as in artificial lights, respectively, and there was a nonsignificant difference (*p* > .05) in opinion between the two raters. The reliability between the two raters in samples of the family Stegodontidae was determined as perfect agreement, *K* = 0.860 and *K* = 0.817 in artificial as well as natural lights, respectively. Similarly, there was a nonsignificant difference (*p* > .05) in the opinion of the raters. The reliability of the family Elephantidae also showed perfect agreement (*K* = 0.914 and *K* = 0.932) between the opinions of two raters in both artificial and natural light, respectively, with non significant (*p* > .05) differences.

### 3.6 Comparison of stress between families and genera

The comparison for the occurrence of frequency of EH between all the **4** Siwalik families, as well as **9** genera of the Order Proboscidea, included in this framework, was analyzed statistically. The normality of the data is checked by the Shapiro–Wilk test. Here the *p*‐values (<.05) revealed that the data was nonparametric. So, the data was analyzed through the Kruskal–Wallis test by using SPSS ver‐21. The comparison among these **4** families and **9** genera was done, defining maximum and minimum range. The *p*‐value (*p* = .105 and *p* = .254) was nonsignificant (*p* > .05) for each group, respectively.

In pursuit of multiple comparisons between each of the families and the genera Mann–Whitney *U* test was performed after the Kruskal–Wallis test. The comparisons of significant level (*p*‐value) for the occurrence of EH within **4** families and **9** genera were individually given in the subsequent Tables 4 and 5. The comparison between all the four Siwalik families showed that the families Deinotheriidae, Gomphotheriidae, and Stegodontidae are significantly (*p* < .05) different from the family Elephantidae, whereas, the other comparisons have nonsignificant (*p* > .05) differences. This comparison indicated that the family Elephantidae has significantly less chances for the occurrence of EH and was more stable. The detail of family comparisons along with *p*‐values is given below in Table 4. Similarly, the detail of comparisons at the generic level is also given below in Table 5.

**TABLE 4:** *p*‐Value comparison table analyzed by the application of Mann–Whitney *U* test on the Siwalik families for the occurrence of EH.

Siwalik families	Gomphotheriidae	Stegodontidae	Elephantidae
Deinotheriidae	0.572	0.717	0.019
Gomphotheriidae	–	0.823	0.036
Stegodontidae	–	–	0.027

**TABLE 5:** *p*‐Value comparison table analyzed by the application of Mann–Whitney *U* test of the Siwalik genera for the occurrence of EH.

Siwalik Genera	*Gomphotherium*	*Anancus*	*Protanancus*	*Choerolophodon*	*Stegolophodon*	*Stegodon*	*Palaeoloxodon*	*Elephas*
*Deinotherium*	0.731	0.559	0.414	0.271	0.830	0.626	0.364	0.022
*Gomphotherium*	–	0.766	0.315	0.195	0.891	0.445	0.295	0.16
*Anancus*	–	–	0.263	0.181	0.677	0.363	0.248	0.020
*Protanancus*	–	–	–	0.954	0.350	0.620	0.746	0.342
*Choerolophodon*	–	–	–	–	0.218	0.527	0.690	0.196
*Stegolophodon*	–	–	–	–	–	0.506	0.321	0.18
*Stegodon*	–	–	–	–	–	–	0.489	0.060
*Palaeoloxodon*	–	–	–	–	–	–	–	0.760

## 4 DISCUSSION

Enamel hypoplasia observed in proboscideans depends on the developmental timings of tooth crowns in these animals. Modern proboscideans lack permanent cheek teeth, which succeed one another by an unusual horizontal tooth displacement mechanism. This mechanism occurs only in elephanti‐morph proboscideans (gomphotheres, Stegodontids, Mammoths, and Elephants). Horizontal tooth displacement provides elephanti‐morphs with an adaptive advantage over primitive proboscideans (Deinotheriids) with vertical tooth replacement (Sanders, 2017). This study included all the linear defects manifested as thin bands of defective enamel, which unlikely seem to be a general nutritional stress. This chapter emphasizes the occurrence of the anomaly (EH) and its correlation to the fluctuating palaeoecological conditions and vegetational patterns, linked to the movement of proboscideans out of the Siwaliks.

### 4.1 Comparison of EH in the family Deinotheriidae

The Deinotheriids represent different morphological traits as compared to the other proboscidean lineages. The smaller species *D. pentapotamiae* has higher incidences of EH (32%) compared to the larger size *D. indicum* (18.52% EH), supposed to compete with more or less resources situated in the reach of smaller herbivores like, *Listriodon pentapotamiae* which have nearly the same dental structure (Samiullah et al., 2010). Both of the *Deinotherium* species are coeval in the Siwaliks of Pakistan with similar niche and diet patterns, competition may also be a major role to acquire more diet related environmental stress. Srivastava et al. (2018) reconstructed the Middle Miocene climate and vegetation on the basis of palaeo flora (13–11 Ma) at Surai Khola section, Nepal. The mean annual precipitation (MAP) was quite the same, but the vegetation shifted from evergreen to deciduous forests towards the Late Miocene with occasional forest fire (Karp et al., 2018). The Deinotheriids were the most affected animals compared to other browsers as they are almost fully reliant on C_3_ vegetation at that time. The diversity of many artiodactyls also decreased after 13 Ma ago (Barry et al., 1995) at the terminal of the Middle Miocene near the Late Miocene. The changing climatic conditions may limit the niche and diet, and cause more competition between species the of same as well as in different taxa with similar diets, leading to an increase in the likelihood of exclusion (Behrensmeyer & Barry, 2005). Furthermore, the Deinotheriids showed pronounced brachydonty, which further support the hypothesis that these animals were dedicated browsers (Harris, 1975; Huttunen, 2002a, 2002b). The stress pattern may also be indicative of increased competition with contemporary mammals. Multiple proxies have already been utilized for the assessment of the diet and ecology of animals. The dental characters, craniometrics, stable isotope analysis, mesowear analysis, and tusk orientation indicated a browsing behavior of the Deinotheriids under a semi closed forestland habitat (Cerling et al., 1999; Cerling et al., 2015; Sarwar, 1977). As the stress was somehow greater in *D. pentapotamiae*, they disappeared earlier than *D. indicum*, perhaps their shorter body size and failure to compete with contemporary herbivores. The post‐*Sivapithecus* interval marks the extinction of many forests inhabiting species therefore, we can assume that the localized extinction of Deinotheriids was also occurred in this interval. The Deinotheriids from Africa and India (Patnaik et al., 2019) also were dominant browsers and preferred the shady trees under an annual rainfall regime (Cerling et al., 1993; Nelson, 2005). The fossil material evaluated and examined in the draft (Figure 1) covers a wider geographical area from Jhelum to Attock (Chinji Formation) of northern Pakistan. Stable conditions and diversified fauna of the Middle Miocene (Manchar Formation) were reported by Raza et al. (1984). The climate consistency decreased substantially after 13 Ma, on the basis of δ^18^O amplitudes, but there was remarkable warming environmental conditions at 10.8 Ma (Holbourn et al., 2013). The Siwalik record imply a reduction of annual rainfall with a change in seasonality of precipitation between savannah (C_4_ grasses) and dry woodlands and monsoon forest (C_3_) was maintained by a small differences during Late Miocene (Badgley et al., 2008). However, the disappearance of the genus and one of its diet competitors, *Listriodon pentapotamiae*, indicate somewhat short but severe environmental changes in the Siwalik region at the terminal of the middle Miocene.

The presence of the *Deinotherium* in the Late Miocene of Europe (Haiduc et al., 2018; Van der Made et al., 2006) and even the Pleistocene of Africa (Fernandez & Vrba, 2006; Geraads, 2010; Maclnnes, 1942) is the one hypothesis that supports the dispersal and migration of this taxon from the Siwalik because of massive environmental change locally. Hence, it can be proposed that 10.8 Ma approx. Was a time of considerable change in regional climate that may have caused reduction in suitable habitats for Deinotheriidae, which have increased the likelihood of migration/extinction of the genus along with many other reasons from the Siwaliks of Pakistan.

### 4.2 Comparison of EH in the Family Gomphotheriidae (published data, included only for comaprisons)

The results of EH from the family Gomphotheriidae allow us to trace the palaeoecological conditions of the Siwaliks. *Gomphotherium* and *Protanancus* are the most primitive genera with less advanced lophodont teeth and presence of lower incisors. The loss of lower incisors in Proboscidea is one of the conditions being considered an evolutionary novelty. The Late Miocene has experienced dry environmental conditions with enhanced seasonality (Herbert et al., 2016). Between 8.5 and 6.0 Ma (Late Miocene), C_4_ savannahs replaced C_3_ forests in the Siwaliks. Few lineages survived (most of the artiodactyls, perissodactyls, primates, and rodents), but several others disappeared during this vegetational transition (Badgley et al., 2008; Morgan et al., 2009). The simplest dental structure of *Gomphotherium* and *Protanancus* from all the gomphotheres indicate that they were browsers on a less hard diet (Fox & Fisher, 2001; Perez‐Crespo et al., 2020). This long term climatic forcing of vegetational structure from forests to grasslands during the Late Miocene was responsible to limit the food resources for these two proboscidean lineages.

The lineage, *Choerolophodon* experienced 16.36% of EH, similar to *Protanancus* (15.79%), lowest in gomphotheres. The genus has no lower incisors and is supposed to be more advanced than *Protanancus* and *Gomphotherium*. *Choerolophodon* survived throughout the Miocene to the Early Pliocene (~5.3 Ma) of the Siwaliks. These mammals somehow succeeded in shifting their feeding pattern toward mixed feeding. The disappearance of *Choerolophodon* by the Early Pliocene at the end of the Late Miocene was because of some large‐scale climatic changes in the Siwalik region. The ecological change during 5.3 Ma demonstrated the stages of C_4_ events, coincident with the Late Miocene–Pliocene boundary (Hynek et al., 2012). Miocene–Pliocene was also a tectonic stress event (Sperber et al., 1989) along with vegetational change towards the expansion of grasslands and intensified global cooling (Behrensmeyer et al., 2007; Liu & Jacques, 2010). The episodes of monsoon intensification were also determined by Sanyal et al. (2004). Despite of increased hypsodonty in the taxon over time, this species failed to compete with more dedicated grazers like bovids during the expansion of C_4_ grasslands.

In Asia, the first records of the anancine gomphotheres are represented by *A. perimensis* from the Potwar Plateau, Pakistan, and Perim Island, India. From Pakistan, *A. perimensis* is documented from the Dhok Pathan Formation (DPF) dated back between 9.8 and ∼3.5 Ma (Barry et al., 2013; Tassy, 1983). *Anancus* are reported from 8.6–8.1 Ma (Flynn et al., 2013; Patnaik, 2013). Tobien (1978) and Tassy (1983) developed its correlation to the DPF, whereas Pickford and Pourabrishami (2013) correlated it to the European middle Turolian (MN12). Further, towards the east, the first record of *Anancus* in China is traced toward the end of the Baodean Land Mammal Stage (∼7.1–5.3 Ma) (Qiu et al., 2013). *Anancus* went extinct from the Siwaliks during the Middle Pleistocene (1.6 Ma). The high number of affected teeth of *Anancus* with EH indicated that the members of this lineage lived under intense environmental conditions. The mid‐Pleistocene climatic transition profoundly affected the distribution and evolution of both plant and animal communities with prevailing ice sheets (Head et al., 2008). The average global long term ice volume increased between 1.25 Ma and 700 ka associated with Major cooling phases (Clark et al., 2006). The mass death of these proboscideans was also reported by Asevedo et al. (2012) during the Pleistocene because of extended periods of low humidity with mixed oak forests of a dry climate dominated by open vegetation (Ravazzi & Strick, 1995). These very unstable ecological conditions, which started to occur in the Early Pleistocene, followed by substantial glaciation with ice sheaths and cooling regimes during the Middle Pleistocene (1.8–1.7 Ma), may also played a vital role to deal with *Anancus*. Smith and DeSantis (2020) uncovered that gomphotheres faced ecological displacement because of the reduction of closed forests to open grasslands during the Pleistocene. The current study concluded that this clade was severely affected by two spells of palaeoenvironmental change; during the Late Miocene and at the Middle Pleistocene of the Siwaliks.

### 4.3 Comparison of EH in the family Stegodontidae

The detail of the occurrence of EH was given in Table 2. The analyzed material belonging to the family Stegodontidae ranges from the Middle Miocene to the Early Pleistocene (12.5–2.3 Ma), given in Figure 3. The *Stegolophodon stegodontoides*, the Late Miocene species of early Stegodontidae, and *Stegodon ganesa*, a Middle Pleistocene species of the late Stegodontidae exposed to greater amounts of EH anomaly. The other Stegodontidae also have a notable amount of EH.

The Late Miocene massive global changes reported by Herbert et al. (2016) had affected the large areas of terrestrial environments and ecosystems, including subtropics, and experienced drying and enhanced seasonality. The longer drying and cooling regimes triggered the reconstruction of terrestrial plant and animal communities in both hemispheres, caused the development of the Sahara Desert (Schuster et al., 2006) and the radiation of some succulent plant lineages (Arakaki et al., 2011). This modification in vegetational pattern is because of long term monsoonal changes in the Siwalik sub‐region during Late Miocene and natural disasters as well. The expansion of savannas replacing the tropical and subtropical forests is a characteristic of the Late Miocene (Cerling, Harris, Ambrose, et al., 1997; Cerling, Harris, MacFadden, et al., 1997). The Late Miocene of Himalayas foreland shifts from dominant vegetation of shrubs and trees towards open C_4_ grasslands with a long term climatic drying after 7.7 Ma ago (Clift et al., 2020; DeMiguel et al., 2019; Merceron et al., 2004). This drying is also closely linked to global cooling (Liu & Jacques, 2010). A rapid radiation and diversification was reported by Arakaki et al. (2011), first during 8–6 Ma, at Late Miocene times and the second at 3–2.5 Ma of Plio‐Pleistocene times.

*S. insignis* with very low magnitude (15.79%) of EH revealed that the Pliocene environmental conditions were slightly more stable than the previous epochs. The supposition got more strength by the recurrence of *Deinotherium* fossils (almost purely browsers) from the Pliocene of India (Colbert, 1935). Highly increased magnitude of EH in *S. ganesa* (33.33%) revealed that the Pleistocene of the Siwaliks (2.58–0.6 Ma) had very unstable palaeoenvironmental conditions with much dominant C_4_ environment and cooling episodes (Clark et al., 2006; Head et al., 2008; Ravazzi & Strick, 1995; Smith & DeSantis, 2020). The conditions were unsuitable for *S. ganesa*, the latest survivors of the family Stegodontidae, profoundly feeding on C_3_ plants. Morgan (1973) suggested that a cold period prior to 43,000 years ago may have caused the elimination of trees. This elimination of trees, the reduction of habitats, and the mean average temperature with the expansion of grazing climatic conditions was not suitable for Stegodontids. The improved dental structures of the *Stegodon*, compared to the other co‐existed Mastodonts, was also a major tool to proliferate their occurrence from the Miocene–Pliocene boundary, to the Pleistocene. The severe and hasty Pleistocene climate crumbled their endurance at its terminal socket more or less before 1.6 Ma ago from the Siwaliks of Pakistan. Some of them may have migrated to the nearest stable habitats. However, the changes were too severe to take time for evolution.

### 4.4 Comparison of EH in the family Elephantidae

The analyzed material of the family Elephantidae ranges from the Pliocene to the Middle Pleistocene of the Siwaliks. Many researchers (Barry et al., 1982; Barry et al., 2002; Dennell et al., 2006; Hussain et al., 1992; Nanda, 1997; Shah, 2009) estimated the range zone of *Elephas* in the Siwaliks of Pakistan as 2.9–1.5 Ma for *E. planifrons* and 2.7–0.6 Ma for *E. hysudricus* (Hussain et al., 1992; Nanda, 1997). The *Palaeoloxodon*, a giant immigrant, appeared during the Pleistocene (1.8 Ma) of the Siwaliks.

A very less occurrence of EH 8.47% in elephants compared to the other proboscideans can be correlated with the shape of teeth. The increased complexity of molar teeth in elephantids by the presence of higher ridge plates (more than 25 in number) and increased hypsodonty index indicate their adaptations to the open C_4_‐ environments (Patnaik et al., 2019). Both lineages of the family were extinct until the Late Pleistocene of the Siwaliks. In different studies, hypsodonty index (HI), number of ridge plates, micro and mesowear analysis, EH analysis, and the composition of stable isotopes carbon δ^13^C and oxygen δ^18^O are correlated to the proportion of the preferred diet, vegetational patterns, and the intake of water (Damuth et al., 2002; Eronen et al., 2010; Janis, 1993; Kaiser et al., 2013; Patnaik, 2015; Patnaik et al., 2019; Roohi et al., 2015). The studies indicate that the Pleistocene elephants (immigrants into the Siwaliks) were pure C_4_ grazers. These elephants can be considered among the earliest large mammals who adapted purely towards the C_4_ vegetational type along with equids (Polissar et al., 2019; Quade et al., 1992). The Late Pleistocene was the time of great climatic variations with less humid to colder and drier environments accompanied by prevailing ice sheaths (Clark et al., 2006; Head et al., 2008; Ravazzi & Strick, 1995; Smith & DeSantis, 2020).

The current results of EH indicate that the elephants are the successful grazers of the Savannah environment and have shifted their diet from C_3_‐ plants to C_4_‐ grasses. This change of diet is the result of a continuous struggle of individuals and the loss of many ancestral proboscidean lineages. The multiple occurrences of EH in Elephantids indicate short term severe palaeoenvironmental changes in the Siwaliks of Pakistan. Conversely, some members become resilient in these fluctuations but faced continuous reduction due to mortality, migration as well as evolution. The evolution and appearance of high ridge plated dentitions with maximum complexity of lophodonty in these elephants was in response to the fluctuating environmental and climatic conditions. This fluctuating environment put pressure on to the Plio‐Pleistocene elephantids and resulted in the evolution of the living Asian species, *E. maximus*, with maximum adaptation to the subtropical arid conditions with C_4_‐ environment.

**FIGURE 7:**
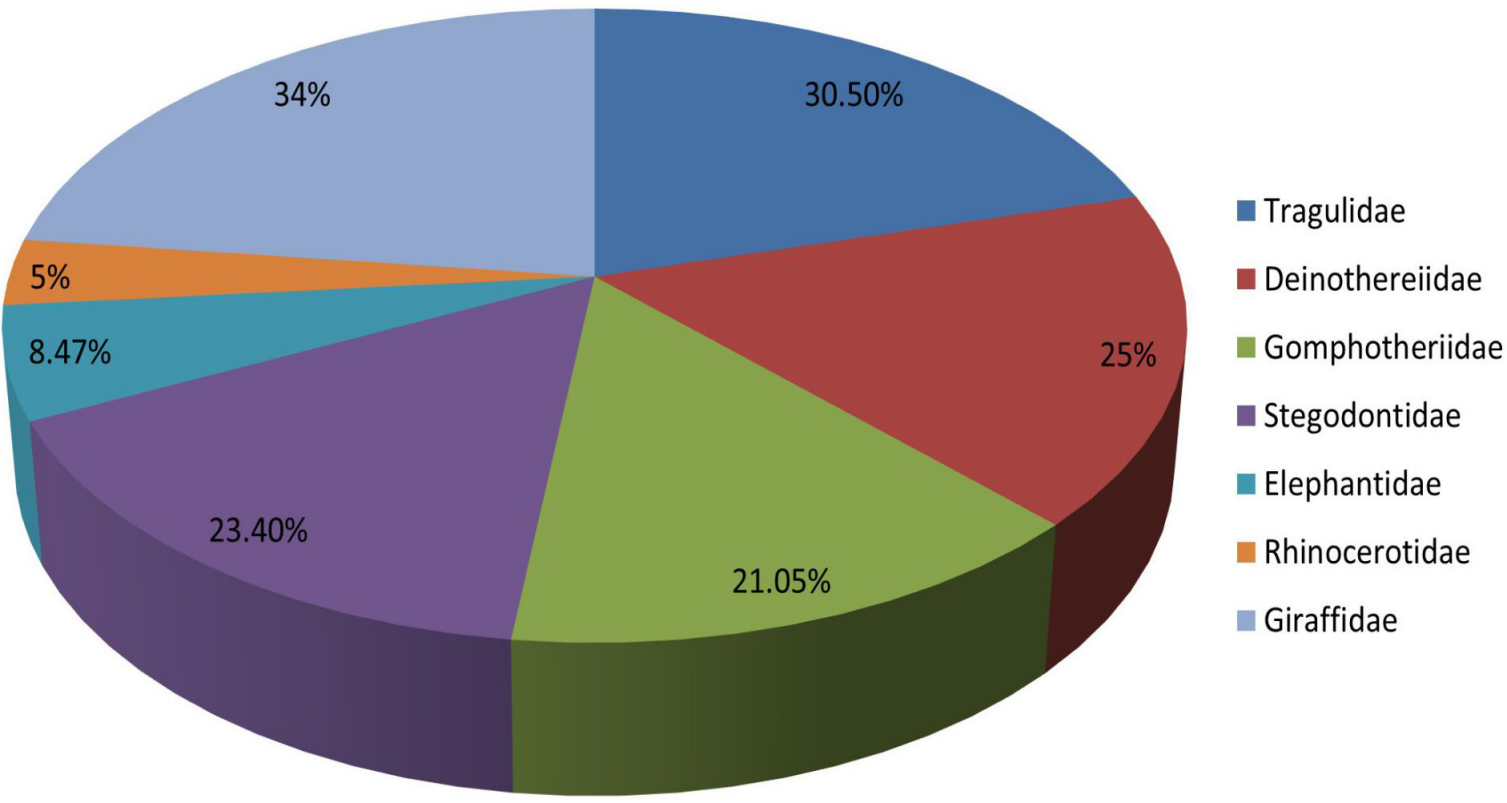
Comparison of the occurrence of stress as EH in the studied Siwalik mammalian families. Giraffidae and Tragulidae (Ahmad et al., 2018, 2020), Rhinocerotidae (Roohi et al., 2015).

**FIGURE 8:**
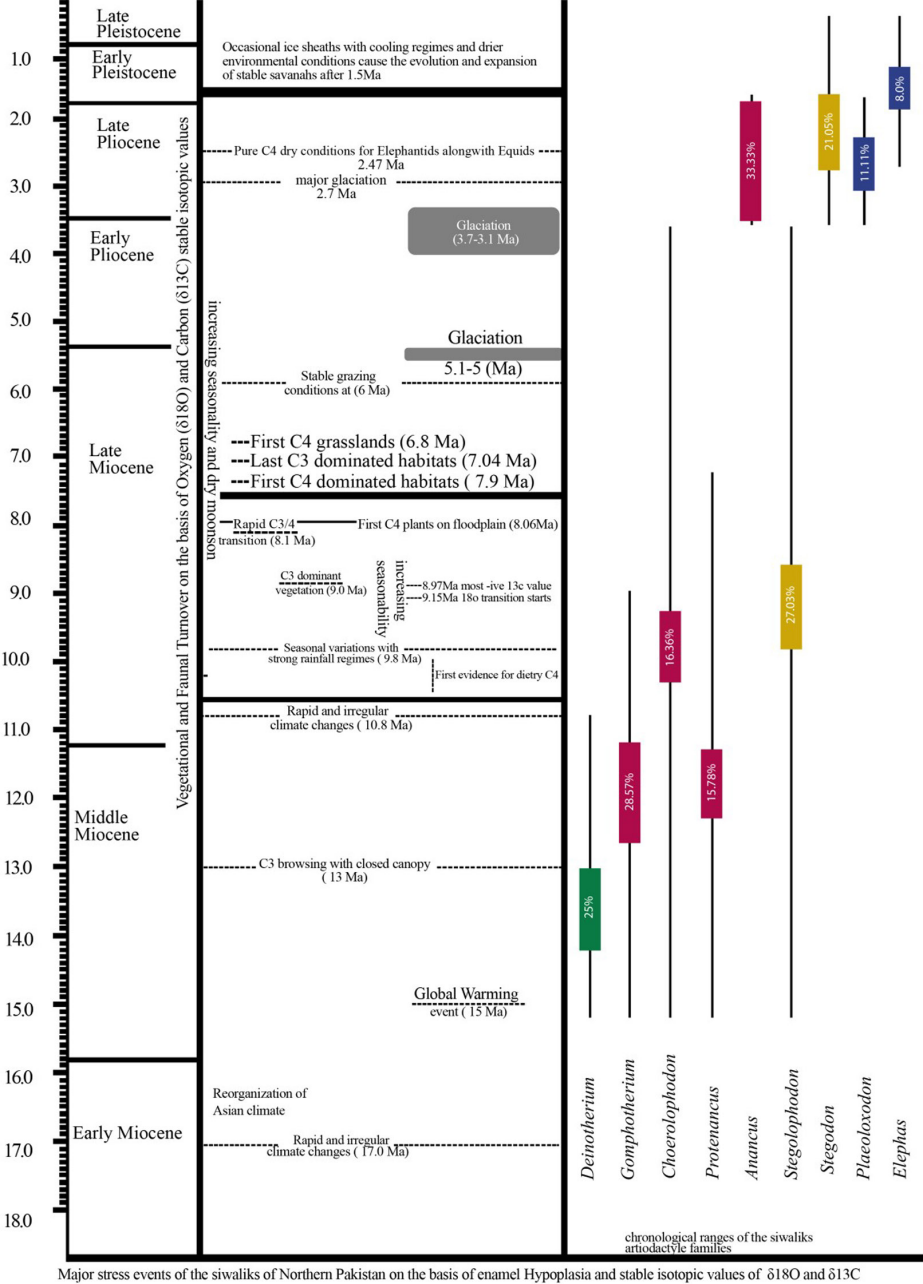
Genus level comparison of the Siwalik stress events on different proboscideans, each color indicates a different family (green = Deinotheriidae, red = Gomphotheriidae, yellow = Stegodontidae, blue = Elephantidae); length of the color bars indicate the frequency of environmental stress during different time intervals of the Siwaliks palaeoenvironment (Barry et al., 2002; Barry & Flynn, 1990; Dennell et al., 2006).

The comparison of present results of EH in Proboscidea with previously published data on the other Siwalik mammals is given in following Figure 7. The occurrence of EH in different Siwalik mammal families indicate that the ecological stress is supposed to be a major contributor for the evolution and migration of many taxa from the Siwalik sub‐region. Similarly, the Figure 8 also indicated that the animal taxa, with less stress, survived longer by the evolution of more advanced dental and morphological characters in the Siwaliks compared to the others. The current results suggest that the elephantids were not extinct from the Siwaliks but evolved to the present Asiatic genus “*Elephas*” still present in the Indian subcontinent (*E. maximus*).

## 5 POTENTIAL LIMITATIONS OF MICROTOMOGRAPHY

Microtomography is used to obtain a high resolution of a physical object without destroying the original object that can't be obtained by any other nondestructive technology. It can be used to study the interior structure of the tooth and other biological samples without having to cut the samples, preserving the samples or specimens for future studies.

Actually, I didn't find the facility of Microtomography for such type of study anywhere in Pakistan yet. The facility is only available in hospitals for disease diagnostics, but they didn't agree to run it on our samples scanning due to possible contamination of the instruments. In addition, sending samples to foreign countries for analysis is also very laborious, expensive, and difficult due to COVID. Hence, I will keep trying to find out, but it still looks impossible at this point. I would try my best to use this technique in future work.

## 6 CONCLUSIONS

The current analysis allowed us to estimate that the Siwalik proboscideans preferred moist and warm climatic regimes with predominantly a browsing habitat similar to their ancestors, as proposed by Liu et al. (2008) for *Barytherium* and *Moeritherium* in Fayum, Egypt. The frequency of EH is linked to the changes in ecological conditions related to the vegetational pattern and competition with other herbivore communities of an area between the Middle Miocene and to Pleistocene of the Siwaliks.

As enamel hypoplasia primarily reflects episodes of systemic stress which can weaken the immune system of animals, reduced fitness, and cause failure to survival, causes mortality at a big scale during younger ages (Temple, 2014), supposed to be a one of many contributing factors to increase the likelihood of extinction (Barrón‐Ortiz et al., 2019). Proboscideans are social animals (Hacker et al., 2015). Sociality can include increased feeding competition (Silk, 2007), greater likelihood of disease transmission (Gompper, 1996), loss of reproductive fitness (Armitage, 1999), intra‐species aggression (Whitehead, 1997), and increased detection by predators or prey (Beck et al., 2012). This hypothesis supports that proboscideans experienced increased levels of systemic physiological, ecological, predatory, and competitive stress, which can be linked to the reduced dietary resources, specifically during the Late Miocene and Pleistocene times. This enhanced competition for survival triggered the likelihood of complete migration of these herbivore animal taxa from the Siwaliks. These constraints were more pronounced during the Latest Miocene and Pleistocene times. Figure 8 provides the correlation and comparisons of tooth EH to the faunal and vegetational changes occurring throughout the Neogene of the Siwaliks.

We believe that for a better understanding of the dietary regime utilized by the proboscideans, a comprehensive microwear and mesowear, along with stable isotopic analyses from proboscidean tooth enamel, is further required in the future.

## AUTHOR CONTRIBUTIONS

**Rana Manzoor Ahmad:** Formal analysis (supporting); writing – review and editing (supporting). **Muhammad Umar Ijaz:** Resources (supporting); software (supporting); visualization (supporting). **Muhammad Imran:** Formal analysis (supporting); investigation (equal); methodology (supporting).

## CONFLICT OF INTEREST

The authors declare no conflict of interest in this study.

## Data Availability

The data used in this article come from PhD dissertation of one of us (Dr. Muhammad Ameen), which will be available at: https://doi.org/10.5061/dryad.qbzkh18jt.
